# An empirical method for slope mass rating-Q_slope_ correlation for Isfahan province, Iran

**DOI:** 10.1016/j.mex.2020.101069

**Published:** 2020-09-19

**Authors:** Mohammad Azarafza, Shahrzad Nikoobakht, Jafar Rahnamarad, Fariba Asasi, Reza Derakhshani

**Affiliations:** a Department of Civil Engineering, Hacettepe University, Ankara, Turkey; b Department of Geology, Yazd University, Yazd, Iran; c Department of Geology, Zahedan Branch, Islamic Azad University, Zahedan, Iran; d Department of Mining Engineering, Urmia University, Urmia, Iran; e Department of Geology, Shahid Bahonar University of Kerman, Kerman, Iran; f Department of Earth Sciences, Utrecht University, Utrecht, Netherlands

**Keywords:** Empirical relationship, Geomechanical classification, Slope mass rating, SMR, Q_slope_, Rock slope engineering, Slope stability, Rock slope classification

## Abstract

The presented article provides an empirical method on rock slope classification, slope mass rating (SMR), Q_slope_, stability condition, failure type and stabilisation procedures for 35 road/railway discontinuous rock slopes after field surveys in Isfahan Province of Iran. Also, it presents the empirical correlation for SMR and Q_slope_ classification system that prepares a link between the stability status (safety factor, reliability condition) and stabilisations (failure mechanism, support system) which performed on natural/trench slopes cases related sedimentary rocks cuts in the studied region. As results, the SMR-Q_slope_ equation for Isfahan Province obtained as SMR = 11.89 ln(Q_slope_) + 71.92 (R^2^ = 0.756).

• This method can be useful on a stability assessment and providing appropriate stabilisations for the discontinuous rock slope based on simple assumptions where used in different geotechnical projects such as road/railway slope, excavations, open-pit mining, trench boring, etc.

• This method can be useful for quick calculation of stability conditions and suggestion of slope maintenance system in a short time as preliminary reactions.

• This method can be used as an effective way to convert SMR and Q_slope_ equations and used both benefits in geo-engineering application faced with discontinuous rock masses.

• This method can be useful for future research on the empirical geomechanically classification and rock mass preliminary quantifications.

• This method can be used as an appropriate database for SMR and Q_slope_ classification.

Specifications Table

**Table:** 

Subject Area:	Earth and Planetary Sciences
More specific subject area:	Rock slope classification
Method name:	Empirical correction of SMR-Q_slope_ relationship for Isfahan province, Iran
Name and reference of original method:	Original method name: SMR Romana M., Serón J.B., Montalar, E., 2003. SMR Geomechanics classification: Application, experience and validation. In: 10th Congress of the International Society for rock mechanics, ISRM 2003–Technology roadmap for rock mechanics, South African Institute of Mining and Metallurgy, 1–4. Romana, M., Tomás, R., Serón, J.B., 2015. Slope Mass Rating (SMR) Geomechanics Classification: Thirty Years Review. In: 13th ISRM International Congress of Rock Mechanics, 10–13 May, Montreal, Canada. Azarafza, M., Akgün, H., Asghari-Kaljahi, E., 2017. Assessment of rock slope stability by slope mass rating (SMR): a case study for the gas flare site in Assalouyeh, South of Iran, Geomech. Eng. 13, 571–584. https://doi.org/10.12989/gae.2017.13.4.571 Original method name: Q_slope_ Bar, N., Barton, N., 2017. The Q-slope method for rock slope engineering, Rock Mechanics and Rock Engineering, 50, 3307–3322. https://doi.org/10.1007/s00603-017-1305-0 Azarafza,M., Ghazifard, A., Akgün, H., Asghari-Kaljahi, E., 2017. Application of the Q-slope classification system for slope stability assessment of the south flank of the Assalouyeh anticline, South Pars Zone, J. Geotech. Geol., 13, 82–90. Azarafza, M., Nanehkaran, Y.A., Rajabion, L., Akgün, H., Rahnamarad, J., Derakhshani, R., Raoof, A., 2020. Application of the modified Q-slope classification system for sedimentary rock slope stability assessment in Iran, Eng. Geol. 264, 105349. https://doi.org/10.1016/j.enggeo.2019.105349
Resource availability:	There are no special resources and field investigation data is presented within the article.

## Method details

The presented article describes the integrated aspect of rock mass rating (SMR) and Q_slope_ systems (SMR-Q_slope_) methods which are used for geomechanical classification and quantification of rock mass characteristics. It was used to both benefits, primarily as flexible empirical approaches to rock mass quantifications and investigate the various issues of the discontinuous to provide a suitable description in design applications [1]. Throughout the present investigation, the two geomechanical classifications, SMR and Q_slope_ been applied to Isfahan Province, Iran, which prepared the appropriate database for a primary check on stability status for studied cases. As known, the SMR and Q_slope_ are experimental classification procedures were provided with a fast way to quantify the rock mass condition. The SMR geotechnical classification derives from the basic rock mass rating (*RMR_b_* or *RMR_89_*). It uses four adjustment factors that depend on the geometric relationship between the discontinuities relative orientations, slope topology and the excavation method. SMR index is a comprehensive and widely used rock mass classification for civil engineering, mining and geoengineering projects which is calculated by [2], [3], [4]:

(1) SMR = RMR_b_ + (F_1_ × F_2_ × F_3_)

where RMR is a geomechanical classification developed by Z.T. Bieniawski [5]; *F_1_* depends on the parallelism between the dip directions of the discontinuities (*α_j_*) and the slope (*α_s_*), *F_2_* depends on the joint dip (*β_j_*), *F_3_* depends on the relationship between the slope angle (*β_s_*) and the discontinuities (*β_j_*) dips and *F_4_* is an adjustment factor which depends on the excavation method employed [2] as follow [3]:

(2) F_1_ = (1-sin| α_j_ - α_s_ |)^2^

(3) F_2_ = tan^2^ (β_j_)

(4) F_3_ = β_j_ - β_s_

**Fig. 1:**
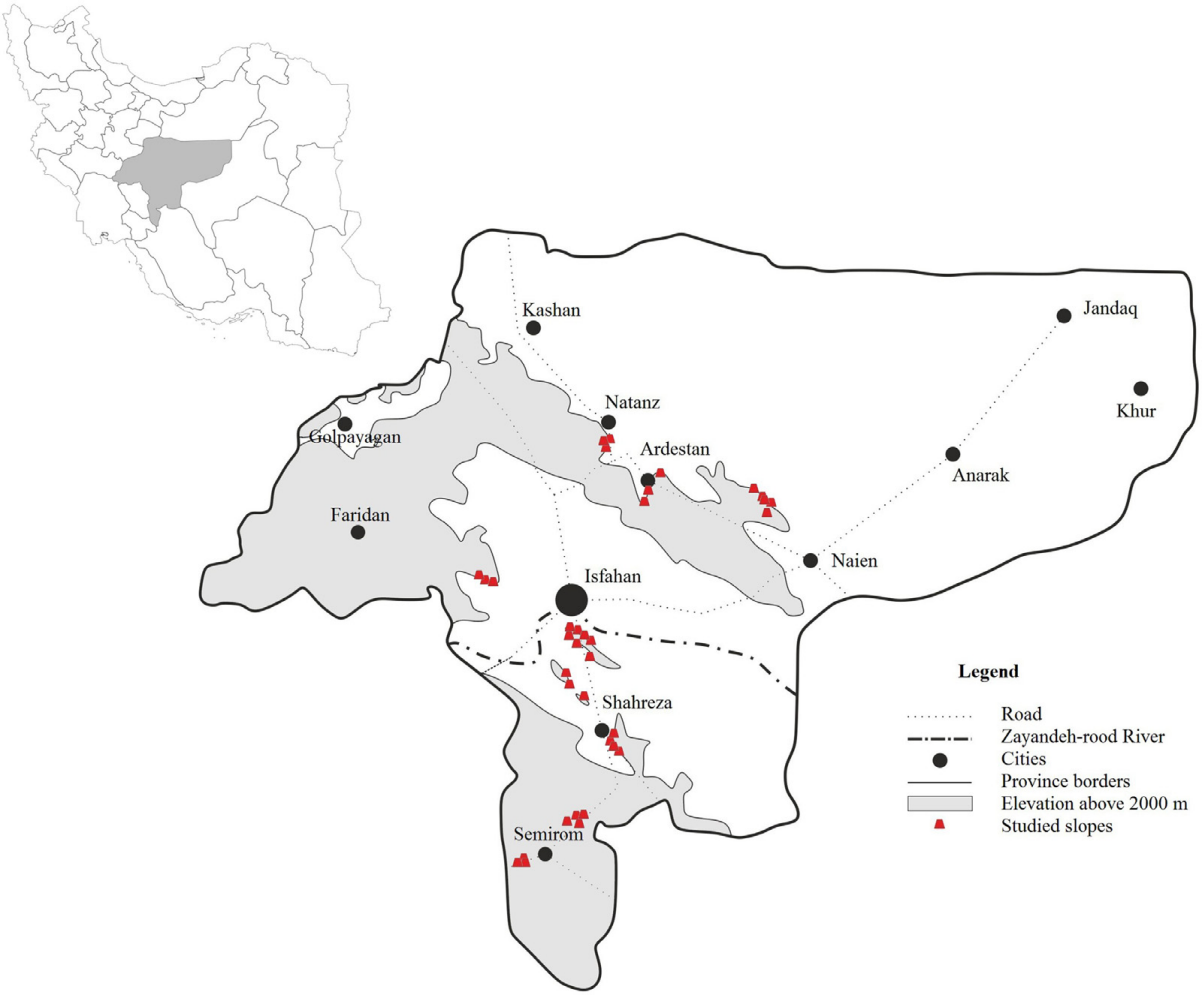
Location of Isfahan Province in Iran.

**Fig. 2:**
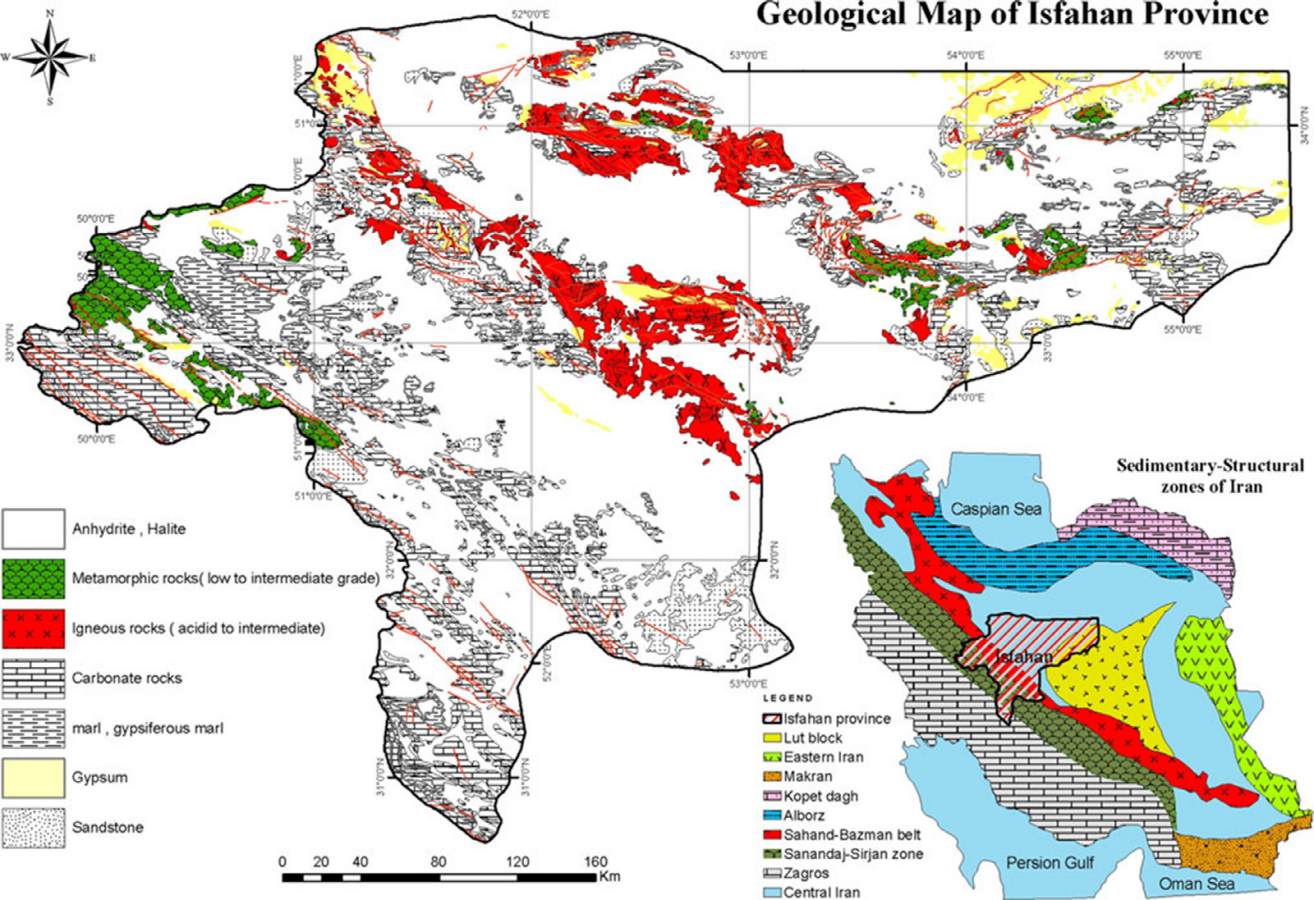
Geological map of Isfahan Province [13].

SMR has been used for over 30 years and provides valuable insight into anticipated slope behaviour[4–5] which provided the experimental aspect of preparing judgements for failure mechanism identification, support system suggestion and stability status for discontinuous rock slopes. In the other hand, the Q_slope_ is an empirical rock slope engineering method for assessing the stability have initially been developed by Bar and Barton [6] which used for quick access to slope stability with minimal assumptions. It is derived from the Q-system which used globally for the characterisation of rock exposures, drill core and tunnels under construction for over 40 years [7], [8]. The Q_slope_ used Q system parameters to slope stability assessments which are modified by some scholar [1] which is calculated using the expression [9], [10]:

(5) Q_slope_ = (RQD/J_n_) × (J_r_/J_a_) × (J_wice_/SRF_slope_)

where *RQD*: rock quality designation, *J_n_*: joint set number, *J_r_*: joint roughness number, *J_a_*: joint alteration number, *J_wice_* and slope relevant strength reduction factors (*SRF*) are applied for long-term exposure to various conditions. The authors are present experimental tables to evaluate the value of each parameter on the field. Table 1 present the advantage of a discrete and integrated aspect of SMR-Q_slope_ methods. Each classification systems have several advantages that can be considered as benefits to the quantification of rock, but the application of a combined issue of these methods can be preparing both of those advantages. In this regard, the SMR-Q_slope_ empirical relationship is presented in this work which used for preliminary stage of stability assessments and reinforcements for discontinuous rock slopes. The article provided data appropriate for the modified SMR-Q_slope_ relationship, which capable of investigating the stability status and providing the appropriate support system for different failure mechanisms. The 35 road/railway slopes cases from Isfahan Province, Iran which are mainly located in sedimentary rocks describe as limestone, marlstone, sandstone and claystone. The studied slopes are required the fast stability assessment, and support implementations have controlled the instabilities in slope bodies. Isfahan province is one of the largest regions in Iran, which is located in the central part of the Iranian plateau. Geologically, in Isfahan province, extensive sequences of sedimentary deposits of metamorphic and igneous rocks of different ages are exposed. Fig. 1 is presented the location of the studied slopes and Isfahan province in Iran and Fig. 2 is given the geological description of the studied region. As seen in the figure, the main sedimentary geological units which belong to Cenozoic and Mesozoic eras [11]. Table 2 is illustrated in the general description of studied cases. Obtaining the data for these cases needed detailed field surveys which are implemented by ISRM instructions and scan-line procedure [12].

**Table 1:** The SMR and Q_slope_ benefits for rock slope data collecting.

Factors	SMR	Q_slope_	SMR-Q_slope_
Stability status	Yes	Yes	Yes
Failure mechanisms	Yes	No	Yes
Support system suggestion	Yes	No	Yes
Rock mass properties	No	Yes	Yes
Discontinuity network	No	Yes	Yes
Quick analysis	No	Yes	Yes
Stress dimension	No	Yes	Yes

**Table 2:** The description for studied slopes dataset.

No.	Characteristics	Description
1	Main lithology	Limestone, marlstone, sandstone and claystone
2	Slope topography	Natural, trenches, excavated
3	Slope height	12 m to 125 m
4	Slope curvature	Flat to rough
5	Slope angle	53° to 90° from natural to trenches
6	Failure types	Wedge, planar, toppling
7	Stability rate (max)	40% stable – 60% unstable (all types included)
8	Involved projects	7
9	Joint density in slope	Low to high
10	Discontinuity orientation	Suitable to unsuitable
11	Seepage	Dry to wet
12	Infill	Mostly clay

Stability analysis and providing appropriate support systems for controlling the instabilities in the discontinuous rock slope is the main task for geo-engineers were faced with different geotechnical projects [14]. But for design appropriate reinforcement systems needed to knowledge about stability condition, failure mechanism, slope mass status, discontinuity network, rock mass geotechnical properties, structural durability, etc. [15], [16], [17], [18]. In the meantime, methodologies that allow for quick analysis with low assumptions have always been considered by professionals especially the empirical geomechanical classification methods especially rock mass rating (SMR) and Q_slope_ systems were found by researchers as a flexible procedure to achieve suitable process in rock slope instabilities [19].

**Fig. 3:**
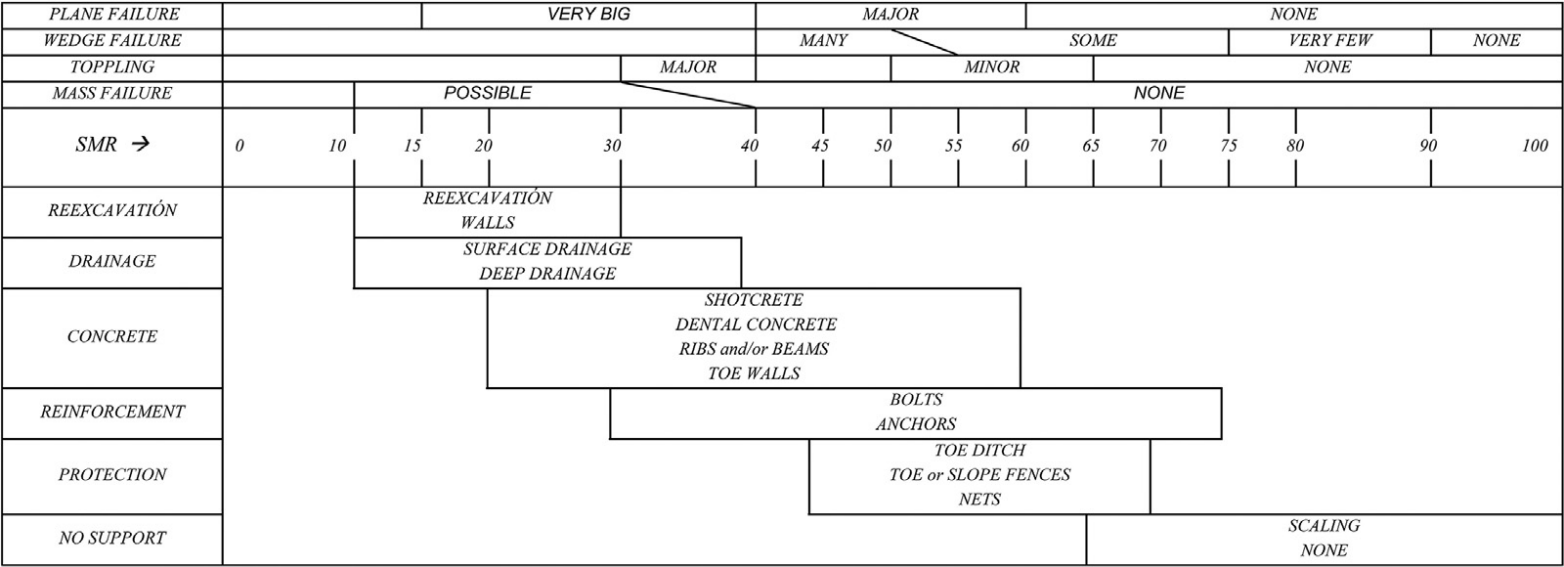
The SMR chart for slope maintenance system [3].

**Fig. 4:**
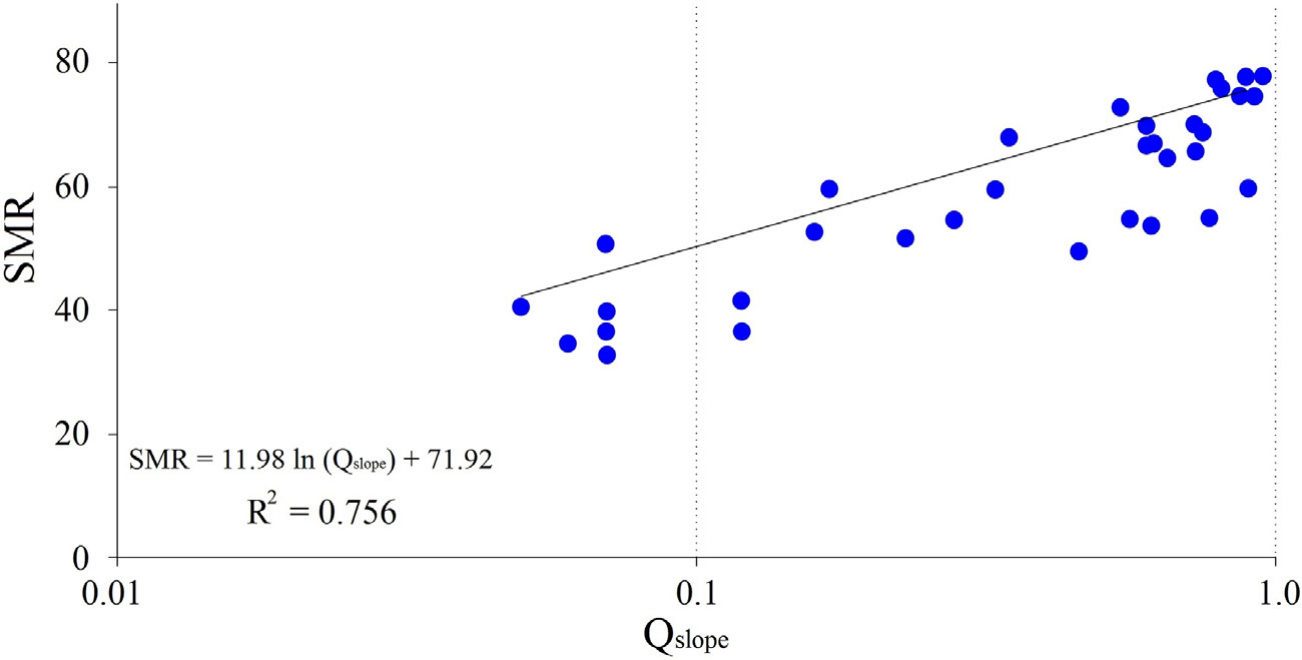
The SMR-Q_slope_ empirical relation chart.

By considering the numerous classification systems developed based on SMR and Q_slope_ systems to provide more detailed and accurate quantifications. In this regard, the scholars attempted to prepare different cases as slope datasets around of the world, preparing the primary stabilisations based on SMR and Q_slope_ classifications [20], [21], [22], [23], [24], [25]. Generally application of the geomechanical classifications for primary slope stability assessment and suggesting the in-situ supporting system can be helpful to prevent the first-time rock failures in different excavation operations [22]. But utilising the appropriate methodology to cover the more uncertainties can be preparing flexible stabilisation [20]. Considering the variability of various elements to provide the SMR or Q_slope_ systems can be used to present the empirical preliminary relationship to use both benefits [19]. The SMR is provided the support system requirement based on the slope conditions and SMR value as well as presented in Fig. 3. By using the results of the Q_slope_ stability number [1,6] and SMR support suggestion can be provided the appropriate maintenance system for slopes. Table 3 presents the SMR and Q_slope_ data for studied slopes. After providing the field investigation processing, the data is categorised and used for optimal line equation estimation based on regression analysis. The obtained SMR-Q_slope_ empirical relationship is presented in Eq. (1). Fig. 4 is given the SMR-Q_slope_ link for studied cases.

(6) SMR = 11.89 ln(Q_slope_) + 71.92

**Table 3:** Data description for SMR and Q_slope_ empirical survey.

No.	Geological Unit	SMR	Q_slope_	Stability condition	Failure type	Stabilisation method
1	Sandstone	75	0.92	Stable	none	none
2	Limestone	76	0.81	Stable	none	none
3	Limestone	60	0.90	Local unstable	Planar	Bolts/Anchors
4	Limestone	55	0.28	Stable	none	none
5	Sandstone	60	0.33	Stable	none	none
6	Claystone	40	0.07	Unstable	Wedge	Shotcrete/Ribs/Beams/Bolts
7	Claystone	66	0.73	Local unstable	Planar	Bolts/Anchors
8	Marlstone	37	0.07	Unstable	Toppling	Shotcrete/Ribs/Beams/Bolts
9	Limestone	55	0.56	Unstable	Wedge	Shotcrete/Ribs/Beams/Bolts
10	Limestone	65	0.65	Local unstable	Wedge	Shotcrete/Bolts/Mesh
11	Limestone	55	0.77	Local unstable	Wedge	Shotcrete/Ribs/Beams/Bolts
12	Sandstone	77	0.80	Stable	none	none
13	Claystone	67	0.60	Local unstable	Wedge	Shotcrete/Bolts/Mesh
14	Marlstone	70	0.60	Stable	none	none
15	Marlstone	53	0.16	Local unstable	Wedge	Shotcrete/Bolts/Mesh
16	Claystone	37	0.12	Unstable	Wedge	Shotcrete/Ribs/Beams/Bolts
17	Sandstone	73	0.54	Stable	none	none
18	Limestone	52	0.23	Local unstable	Wedge	Shotcrete/Ribs/Beams/Bolts
19	Limestone	54	0.61	Local unstable	Wedge	Shotcrete/Ribs/Beams/Bolts
20	Marlstone	68	0.35	Stable	none	none
21	Claystone	35	0.06	Unstable	Planar	Shotcrete/Bolts/Anchors
22	Claystone	33	0.07	Unstable	Wedge	Shotcrete/Ribs/Beams/Bolts
23	Marlstone	78	0.95	Stable	none	none
24	Claystone	41	0.05	Local unstable	Wedge	Shotcrete/Ribs/Beams/Bolts
25	Claystone	70	0.73	Local unstable	Planar	Bolts/Anchors
26	Limestone	41	0.05	Unstable	Wedge	Shotcrete/Ribs/Beams/Bolts
27	Limestone	60	0.17	Stable	none	none
28	Marlstone	78	0.89	Stable	none	none
29	Marlstone	50	0.46	Unstable	Wedge	Shotcrete/Ribs/Beams/Bolts
30	Limestone	42	0.12	Unstable	Wedge	Shotcrete/Bolts/Mesh
31	Limestone	69	0.75	Stable	none	none
32	Limestone	51	0.07	Stable	none	none
33	Limestone	75	0.87	Local unstable	Planar	Bolts/Anchors
34	Limestone	67	0.61	Local unstable	Wedge	Shotcrete/Bolts/Mesh
35	Marlstone	68	0.35	Stable	none	none

The SMR and Q_slope_ allowed estimation of stability status (safety factor, reliability condition) and stabilisations (failure mechanism, support system) in rock slopes. It can be used as an advantage to provide the SMR-Q_slope_ link relationship in the preliminary stage of stability assessment and reinforcements for discontinuous rock slopes (Table 1). In this regard, the presented study tries to prepare an empirical correlation link between the SMR and Q_slope_ classification systems which performed on 35 natural/trench slopes cases related sedimentary rocks cuts in Isfahan Province of Iran (Fig. 1). According to the regression analysis for SMR-Q_slope_ empirical relationship, SMR = 11.89 ln(Q_slope_) + 71.92 with R-squared value is 0.756 was estimated for the area (Fig. 3).

By preparing the comparison between the results of this study and the Jorda-Bordehore and his colleagues were conducted on 57 case studies from Bolivia, Ecuador, Laos, Peru and Spain contain SMR = 7.4219 ln(Q_slope_) + 47.196 with R^2^ = 0.427 can be concluded the both task presenting the a near process trend which indicates the existence of a logical and universal relationship between SMR and Q_slope_.

## Declaration of Competing Interest

The authors declare that they have no known competing financial interests or personal relationships that could have appeared to influence the work reported in this paper.
